# Modeling influenza sequence evolution for vaccination

**DOI:** 10.1186/1753-6561-6-S6-P44

**Published:** 2012-10-01

**Authors:** Taijiao Jiang

**Affiliations:** 1 Institute of Biophysics, Chinese Academy of Sciences, Beijing, China

## 

The rapid evolution of the influenza A virus poses a global challenge to public health. Recent events, such as the spread of highly pathogenic H5N1 influenza virus, have heightened concerns of potential pandemics. Thus, there is an urgent need for a better understanding of influenza virus evolution. Due to the development of high-throughput sequencing technologies, large-scale sequencing of influenza viruses has become routine work in influenza surveillance, and analyses of these large-scale viral sequence data have significantly enhanced our understanding of influenza evolution. However, opportunities remain to extract even more useful information to inform influenza prevention and control strategy. As we know, seasonal influenza prevention and control rely largely on the availability of effective vaccines. However, timely and accurate recommendation of vaccine strains is quite challenging, as evidenced by frequent antigenic mismatches between the recommended vaccine strains and circulating strains.

Our recent work has shown that development of sequence-based computational approaches to modeling the antigenic evolution of the influenza virus holds great promise for more effective vaccine strain recommendation [1,2]. Based on whole-genome information, we have developed a network model to represent each H3N2 virus as a nucleotide-co-occurrence network [1]. The network model effectively captures the evolutionary antigenic features of H3N2 virus at the whole-genome level and accurately describes the complex evolutionary patterns between individual gene segments. Our analyses show that the co-occurring nucleotide modules apparently underpin the dynamics of human H3N2 evolution, and that amino acid substitutions corresponding to nucleotide co-changes cluster preferentially in known antigenic regions of the viral surface protein hemagglutinin (HA). Therefore, our study demonstrates that nucleotide co-occurrence networks represent a powerful method for tracking influenza A virus evolution, and that cooperative genomic interaction is a major force underlying influenza virus evolution. Based on the sequences of HA, the major antigen of influenza virus, we have developed a computational method, denoted as PREDAC, to predict antigenic clusters of influenza A (H3N2) viruses with high accuracy from viral HA sequences [2]. Application of PREDAC to large-scale HA sequence data from H3N2 viruses isolated from diverse regions of mainland China identified 17 antigenic clusters that have dominated for at least one season between 1968 and 2010. By tracking the dynamics of the dominant antigenic clusters, we not only find that dominant antigenic clusters change more frequently in China than in the United States and Europe, but also characterize the antigenic patterns of seasonal H3N2 viruses within China. Furthermore, we demonstrate that the coupling of large-scale HA sequencing with PREDAC can significantly improve vaccine strain recommendation for China.
